# *Catadiscus marielaosornae* n. sp. (Digenea: Diplodiscidae) infecting the intestine of Peters’ thin-toed frog, *Leptodactylus petersii* (Steindachner) (Anura: Leptodactylidae) from the Yahuarcaca Lake System, Amazon River (Leticia, Colombia), with phylogenetic analysis

**DOI:** 10.1007/s11230-025-10250-y

**Published:** 2025-09-11

**Authors:** Kamila Cajiao-Mora, José Rancés Caicedo-Portilla, John H. Brule, Haley R. Dutton, Stephen A. Bullard

**Affiliations:** 1 Southeastern Cooperative Fish Parasite and Disease Laboratory, School of Fisheries, Aquaculture, and Aquatic Sciences, College of Agriculture, Auburn University, Auburn, AL 36849 USA; 2 CIBAV Research Group, Veterinary Medicine School, Universidad de Antioquia, Calle 70 No. 52–21, 050034 Medellín, Colombia; 3 Instituto Amazónico de Investigaciones Científicas, SINCHI, Sede Principal, 910001 Leticia, Colombia; 4 Department of Zoology, School for Environmental Sciences and Development, North-West University, Private Bag X6001, Potchefstroom, 2520 South Africa; 5 Southeastern Cooperative Fish Genetics Laboratory, School of Fisheries, Aquaculture, and Aquatic Sciences, College of Agriculture, Auburn University, Auburn, AL 36849 USA

## Abstract

We describe *Catadiscus marielaosornae* Cajiao-Mora and Bullard **n. sp**. (Diplodiscidae) from the intestine of Peters’ thin-toed frog, *Leptodactylus petersii* (Steindachner) (Anura: Leptodactylidae) from the Yahuarcaca Lake System, Colombia. We assigned our specimens to *Catadiscus* Cohn, 1904 because they have a smooth-surfaced, pyriform body, centrally-constricted ventro-terminal acetabulum that lacks an accessory sucker, pharynx with extramural sacs, oesophageal bulb, and single testis. The new species differs from all but *Catadiscus rodriguezi* Caballero, 1955, *Catadiscus marinholutzi* Freitas and Lent, 1939, and *Catadiscus propinquus* Freitas and Dobbin, 1956 by having a vitellarium that is confluent anteriorly (*vs*. two non-confluent fields). It differs from the aforenamed congeners by having a ventral common genital pore that opens anterodextral to the oesophageal bifurcation, round vitelline follicles that become confluent anterior to the testis, and a 1: 2–3 ratio of acetabulum: testis width. Our 28S phylogenetic analysis recovered the new species sister to *C. marinholutzi*, and Diplodiscidae as paraphyletic, with *Catadiscus* sister to a clade comprising a sequence ascribed to *Chiorchis fabaceus* (Diesing, 1838) Fischoeder, 1901 (Cladorchiidae), and sequences of three species of *Diplodiscus* Diesing, 1836. The species description herein includes several features pertaining to the acetabulum, female genitalia, and excretory system that could comprise useful character states with which to more readily classify and diagnose diplodiscid species and genera. To our knowledge, this is the first record of a digenean infecting Peters’ thin-toed frog, and it is also the first record of any parasite infecting any ditch frog (*Leptodactylus* spp.) from Colombia.

## Introduction

Colombia and Brazil have the highest amphibian species diversity worldwide, with 903 (424 [47%] endemic) and 1244 (846 [69%] endemic) species, respectively (Frost, 2024; Acosta-Galvis, 2025). However, few studies have focused on their parasites, especially in Colombia, and relatively speciose frog genera remain practically unsurveyed for trematode parasites there. For example, and relevant to the present study, nothing has been published about the parasites infecting ditch frogs (or “white-lipped frogs”; *Leptodactylus* Fitzinger) (Anura: Leptodactylidae) from Colombia. *Leptodactylus* comprises about 81 species of neotropical ditch frogs distributed from southern North America (Texas) to southern South America and to the West Indies (Hedges and Heinicke, 2007; de Sá et al., 2014; Gazoni et al., 2021; Frost, 2024). Moreover, recent studies related to parasitism in Colombian amphibians have been focused on haemoprotozoans, fungi, and viruses (Prada-Salcedo et al., 2011; Cotes-Perdomo et al., 2018; González et al., 2021; Flechas et al., 2023).

Campião et al. (2014) listed 289 helminth species from 196 published studies treating 186 amphibian host species. Over half of those reports (55%) were from Brazil but only 2.6% were from Colombia (Campião et al., 2014). From within Colombia, we are aware of records for only 19 helminth species infecting seven anuran species (five endemic; Table 1) of Dendrobatidae Cope, Bufonidae Gray, and Ranidae Batsch (see Uribe-Piedrahita, 1948; Ucrós-Guzmán, 1959; Marinkelle, 1970; Brooks 1976; Esslinger, 1989; Speare, 1990; Goldberg and Bursey, 2003; Sánchez et al., 2010; Bechara-Escudero and Asprilla-Murillo, 2007; Bechara and Vélez, 2010; Copete-Sierra et al., 2013).

**Table 1:** Helminth parasites infecting amphibians in Colombia (*endemic amphibian species)

Helminth	Life stage	Host	Locality	References
**Acanthocephala**				
*Oncicola* sp.	cystacanth	harlequin poison frog, *Oophaga histrionica* (Berthold) (Anura: Dendrobatidae)*	Choco; Caldas	Goldberg and Bursey (2003)
*Polymorphus* sp.	cystacanth	harlequin poison frog	Choco; Caldas	Goldberg and Bursey (2003)
**Nematoda**				
*Porrocaecum* sp.	juvenile	harlequin poison frog; condoto stubfoot toad, *Atelopus spurrelli* Boulenger (Anura: Bufonidae)*	Choco; Caldas	Goldberg and Bursey (2003)
*Physocephalus* sp.	juvenile	harlequin poison frog; condoto stubfoot toad	Choco; Caldas	Goldberg and Bursey (2003)
*Strongyloides* sp.	eggs	harlequin poison frog; Lehmann’s poison frog, *Oophaga lehmanni* (Myers and Daly)*; Cauca poison frog, *Andinobates bombetes* (Myers and Daly) (Dendrobatidae)*	Captive at the Cali Zoo (Valle del Cauca)	Copete-Sierra et al. (2013)
*Cosmocerca parva* Travassos, 1925 (Cosmocercidae)	adult	Santa Rita rocket frog, *Leucostethus fraterdanieli* (Silverstone) (Anura: Dentrobatidae)*	Risaralda	Sánchez et al. (2010)
*Cosmocerca podicipinus* Baker and Vaucher, 1984	adult	harlequin poison frog; condoto stubfoot toad	Choco; Caldas	Goldberg and Bursey (2003)
*Ochoterenella* sp.	adult and microfilaria	cane toad, *Rhinella marina* Linnaeus (Anura: Bufonidae)	“Centro, Occidente y Norte”	Marinkelle (1970)
*Ochoterenella complicata* Esslinger, 1989 (Onchocercidae)	adult	cane toad	Pacific lowlands	Esslinger (1989)
**Cestoda**				
*Cylindrotaenia americana* Jewell, 1916 (Nematotaeniidae)	adult	cane toad	Atlantico; Huila; Caldas	Brooks (1976)
*Ophiotaenia bonariensis* Szidat and Soria, 1954 (Proteocephalidae)	adult	cane toad	Atlantico	Brooks (1976)
**Trematoda**				
*Haematoloechus medioplexus* Stafford, 1902 (Haematoloechidae)	adult	Amazon River frog, *Lithobates palmipes* (Spix) (Anura: Ranidae)	Meta	Uribe-Piedrahita (1948)
*Creptotrema lynchi* Brooks, 1976 (Allocreadiidae)	adult	cane toad	Atlantico	Brooks (1976)
*Gorgoderina diaster* Lutz, 1926 (Gorgoderidae)	adult	cane toad	Huila	Brooks (1976)
*Rauschiella robusta* (Brooks, 1976) Razo-Mendivil, León-Régagnon and Pérez-Ponce de León, 2006 (Macroderoididae)	adult	cane toad	Huila	Brooks (1976)
*Pseudosonsinotrema* chabaudi (Caballero y Caballero, 1969) Sullivan, 1974 (Pleurogenidae)	adult	cane toad	Antioquia	Bechara and Vélez (2010)
*Mesocoelium monas* (Rudolphi, 1819) Teixeira de Freitas, 1958 (Mesocoeliidae)	adult	cane toad	Antioquia; Chocó	Bechara and Vélez (2010); Bechara-Escudero and Asprilla-Murillo (2007)
*Mesocoelium sociale* (Lühe, 1901) Odhner, 1910 (Mesocoeliidae)	–	cane toad	–	Ucrós-Guzmán (1959)

Peters’ thin-toed frog, *Leptodactylus petersii* (Steindachner), the type-host for the new species we describe herein, is endemic to the Amazon River Basin and the Guiana Shield (Gazoni et al., 2021; Frost, 2024). In Colombia, the species has been documented in the departments of Amazonas, Caquetá, Casanare, Guainía, Guaviare, Meta, and Vichada (Acosta-Galvis, 2025; Caicedo-Portilla et al., 2022). Ernst et al. (2007) reported the species exclusively in primary forest in Guyana; however, recent observations in Leticia (Amazonas, Colombia) indicate its presence in disturbed areas and secondary forest, often on bare soil and leaf litter (Caicedo-Portilla et al., 2023). Although this frog is widely distributed across the Amazon, only a few parasites (all nematodes) have been identified and reported from it. Bursey et al. (2001) reported adults of *Cosmocerca brasiliense* Travassos, 1925 (Nematoda: Cosmocercidae) from the intestine of frogs captured in the Madre de Dios River Basin, Peru. Goldberg et al. (2009) reported adults of *Cosmocerca podicipinus* Baker and Vaucher, 1984 (Nematoda: Cosmocercidae) from the intestine as well as juveniles of *Physaloptera* sp. (Nematoda: Physalopteridae) encysted in “stomach” of frogs from Tocantins, Brazil. Nascimento et al. (2013) described adults of *Rhabdias breviensis* Nascimento, Gonçalves, Melo, Giese, Furtado and Santos, 2013 (Nematoda: Rhabditida) infecting the lung of frogs from Pará, Brazil. Oliveira-Souza et al. (2020) reported adults of *R. breviensis* infecting the lung, adults of Cosmocercidae gen. sp. infecting intestine, and juveniles of *Ortleppascaris* sp. (Nematoda: Ascarididae) encysted in liver of frogs from Amapá, Brazil. To our knowledge, this is the first record of a digenean infecting Peters’ thin-toed frog but several congeneric frogs host trematodes, including species of *Catadiscus* Cohn, 1904 (Diplodiscidae) (Table 2) (Campião et al., 2014).

**Table 2:** *Catadiscus* Cohn, 1904 (Digenea: Diplodiscidae) species with associated type host, type locality, and museum type series

*Catadiscus* spp.	Type host	Type locality	Type series	References
*Catadiscus dolichocotyle* (Cohn, 1903) Cohn, 1904 (type)	Ecuador sipo, *Chironius grandisquamis* (Peters) (Serpentes: Colubridae)	Argentina	–	Cohn (1903; 1904)
*Catadiscus cohni* Travassos, 1926	cane toad, *Rhinella marina* (Linnaeus) (Anura: Bufonidae)	Brazil	–	Travassos (1926); Sey (1991)
*Catadiscus pygmaeus* (Lutz, 1928) Freitas and Lent, 1939	paradox frog, *Pseudis paradoxa* (Linnaeus) (Anura: Hylidae)	Venezuela	–	Lutz (1928)
*Catadiscus marinholutzi* Freitas and Lent, 1939	criolla frog, *Leptodactylus latrans* (Steffen) (Anura: Leptodactylidae)	Mato Grosso, Brazil	CHIOC 10546–10555	Freitas and Lent (1939)
*Catadiscus uruguayensis* Freitas and Lent, 1939	criolla frog	Montevideo, Uruguay	CHIOC 10540–10545	Freitas and Lent (1939)
*Catadiscus inopinatus* Freitas, 1941	criolla frog	Mato Grosso, Brazil	CHIOC 15374	Freitas (1941)
*Catadiscus freitaslenti* Ruiz, 1943	military ground snake, *Erythrolamprus miliaris* (Linnaeus) (Serpentes: Colubridae)	São Paulo, Brazil	IB No. 5.572	Ruiz (1943)
*Catadiscus mirandai* Freitas, 1943	Carvalho’s Surinam toad, *Pipa carvalhoi* (Miranda-Ribeiro) (Anura: Pipidae)	Espirito Santo, Brazil	CHIOC 15431	Freitas (1943)
*Catadiscus rodriguezi* Caballero, 1955	South American bullfrog, *Leptodactylus pentadactylus* (Laurenti) (Anura: Leptodactylidae)	Valle de Anton, Panama	CNHE 736; CNHE 1516	Caballero (1955); Lamothe-Argumedo et al. (1997)
*Catadiscus propinquus* Freitas and Dobbin, 1956	Amazon River frog, *Lithobates palmipes* (Spix) (Anura: Ranidae)	Pernambuco, Brazil	CHIOC 21606–21611	Freitas and Dobbin (1956)
*Catadiscus corderoi* Mañé-Garzón, 1958	Lesser swimming frog, *Pseudis minuta* (Günther) (Anura: Hylidae)	Montevideo, Uruguay	–	Mañé-Garzón (1958)
*Catadiscus eldoradiensis* Artigas and Pérez, 1964	criolla frog	Eldorado, São Paulo, Brazil	USP Nos. 562, 266	Artigas and Pérez (1964)
*Catadiscus longicaecalis* Poumarau, 1965	jararaca pintada, *Bothrops neuwiedi* (Wagler) (Serpentes: Viperidae)	Chaco, Argentina	*	Poumarau (1965)
*Catadiscus rochai* Correa and Artigas, 1978	velvet swampsnake, *Erythrolamprus typhlus* (Linnaeus) (Serpentes: Colubridae)	São Paulo, Brazil	USP No. 4421	Correa and Artigas (1978)
*Catadiscus hylae* Incorvaia, 1983	Montevideo treefrog, *Boana pulchella* (Duméril and Bibron) (Anura: Hylidae)	Buenos Aires, Argentina	**	Incorvaia (1983)
*Catadiscus pomaceae* Hamann, 1992	golden apple snail, *Pomacea canaliculata* (Lamarck) (Gastropoda: Ampullaridae)	Corrientes, Argentina	***	Hamann (1992)
*Catadiscus marielaosornae* Cajiao-Mora and Bullard **n. sp.**	Peters’ thin-toed frog, *Leptodactylus petersii* Steindachner (Anura: Leptodactylidae)	Yahuarcaca Lake System, Amazonas, Colombia	USNM 1694133 (type); USNM 1694134–6 (paratypes); 348–351-PA-FCA-UdeA (paratypes)	Present study

*Lote N. 51a–k deposited in the Colección Helmintológica del Instituto Nacional de Microbiología “Carlos G. Malbrán” (now Administración Nacional de Laboratorios e Instituto de Salud) Buenos Aires, Argentina.

**Types claimed to have been deposited in the Museo de La Plata, Argentina, were never actually deposited (Drago, F., personal communication, 2025).

***Deposited in Museo Argentino de Ciencias Naturales “Bernardino Rivadia” Buenos Aires, Argentina (no accession number was provided).

Abbreviations: Coleccion de Parasitología Animal, Departamento de Ciencias Agrarias, Universidad de Antioquia, Medellín, Antioquia, Colombia (PA-FCA-UdeA); Colección Nacional de Helmintos, México D.F., México (CNHE); Departamento de Parasitologia do Instituto de Ciencias Biomédicas da Universidade de São Paulo, Brazil (USP); Instituto Butantan, São Paulo, Brazil (IB); Coleção Helmintológica do Instituto Oswaldo Cruz, Rio de Janeiro, Brazil (CHIOC); Smithsonian National Museum of Natural History, Washington DC (USNM).

*Catadiscus* is interesting because it is the only genus of Diplodiscidae Cohn, 1904 (Digenea: Paramphistomoidea) that, as currently classified, includes species that collectively infect both amphibian and reptile hosts in South America (Table 2) (Jones, 2005a). The only nucleotide sequence available for a species of *Catadiscus* previous to the present study was from Queiroz et al. (2021). Those authors described the life cycle of *Catadiscus marinholutzi* Freitas and Lent, 1939, wherein a planorbid snail shed cercariae that infected several tree frogs (Hylidae Rafinesque) and southern frogs (Leptodactylidae Werner).

We herein describe a new species of *Catadiscus* infecting the Peters’ thin-toed frog from the flooded rainforest habitat of the Yahuarcaca Lake System (Leticia, Amazonas, Colombia) and use representative nucleotide sequences to place it within a phylogeny for Diplodiscidae.

## Materials and methods

### Specimen collection, preparation, and deposition

During an expedition to Leticia, Amazonas, Colombia, on 24 April 2023, the authors collected five adult Peters’ thin-toed frogs from bare soil and leaf litter in the flooded rainforest habitat (4°11′36.7"S, 69°57′17"W; 70 m above sea level) of the Yahuarcaca Lake System. Frogs were euthanized by immersing them in a solution of hydrous chlorobutanol (1,1,1-trichloro-2-methyl-2-propanol), 95% non-denatured ethanol (EtOH), and water, following Simmons (2015). Immediately after, the viscera from each frog were removed intact or examined within the abdominal cavity (immersed slightly in saline). All organs were examined using a Zeiss Stemi 305 stereo-dissecting microscope (Carl Zeiss Microscopy, White Plains, NY). The alimentary canal was opened longitudinally to expose the lumen, and the intestinal mucosa was examined for the presence of parasites. Nineteen trematode specimens were collected from the intestine of four frogs. Fourteen trematode specimens were heat-killed and immediately fixed in 10% neutral buffered formalin (n.b.f.) for morphology. Five specimens were preserved in 95% EtOH for DNA extraction. Fixed specimens were rinsed in water then stained overnight in Van Cleave’s and Ehrlich’s hematoxylins following Cajiao-Mora et al. (2024). Three specimens were drawn using an Olympus BX51 compound microscope (Olympus, Tokyo, Japan) equipped with differential interface contrast optical components and a drawing tube. Measurements from specimens in optimal conditions were obtained using a Jenotipik Gryphax camera (Jenotipik AG, Jena, Germany). Measurements are reported in micrometers (μm), for specimens in optimal ventral orientation, unless otherwise stated as the range followed by the mean in parentheses. Some comparative measurements for species of *Catadiscus* (types are not available for international loan) were estimated from published drawings, when available, for the pharyngeal complex and oesophagus. Specimens for scanning electron microscopy (SEM) were washed with de-ionized water, dehydrated through a graded EtOH series, pipetted onto a 45 micrometer (μm) mesh cut to fit within a 30 μm microporous specimen capsule, critical point dried in liquid CO2, mounted on SEM aluminum stubs with double-sided carbon tape, sputter-coated with gold palladium (19.32 g/cm3; 25 mA), and viewed with a Zeiss EVO 50VP SEM (Warren and Bullard, 2019). Common names for amphibians follow Frost (2024). Nomenclature for Diplodiscidae follow Jones (2005a). Terminology for the digestive system follow Sey (1991) and Jones (2005b) except that we refer as a pharyngeal complex to the pharynx plus pharyngeal sacs. The holotype and three paratypes were deposited in the National Museum of Natural History’s Invertebrate Zoology Collection (USNM, Smithsonian Institution, Washington, DC). Three additional paratypes were deposited in the Animal Parasitological Collection (APC, Agrarian Science Department, Universidad de Antioquia, Medellín, Antioquia, Colombia). Frogs were deposited in the SINCHI Institute Amphibian Collection (Instituto Amazónico de Investigaciones Científicas, SINCHI, Leticia, Amazonas, Colombia).

### DNA extraction

Two EtOH-preserved specimens, from two of the four infected frogs, were used for DNA extraction and sequencing. To perform the extraction the DNeasy™ Blood and Tissue kit (Qiagen) was used following the manufacturer’s protocol, except that the proteinase-K incubation period was extended overnight. After extraction, DNA concentration was measured using a NanoDrop-One Microvolume Spectrophotometer (Thermo Fisher Scientific), diluted to <50 ng/μL, and stored at –20 C. Segments of the 28S and ITS2 were amplified. The partial 28S gene was amplified using the forward primer U178 (5′-GCACCCGCTGAAYTTAAG-3′), and reverse primer L1642 (5′-CCAGCGCCATCCATTTTCA-3′), sequencing primers 300F (5′-CAAGTACCGTGAGGGAAAGTTG-3′) and 1200R (5′-GCATAGTTCACCATCTTTCGG-3′) (Lockyer et al., 2003). The ITS2 region was amplified using forward primer GA1 (5′-AGAACATCGACATCTTGAAC-3′; Anderson and Barker, 1998) and reverse primer ITS2.2 (5’-CCTGGTTAGTTTCTTTTCCTCCGC-3’; Cribb et al., 1998). PCR reactions were performed following Truong et al. (2021). DNA amplification was verified with a 1% agarose gel stained with ethidium bromide. PCR products were purified using the QIAquick PCR Purification Kit (Qiagen) according to the manufacturer’s protocol, except that the last elution step was performed with autoclaved nanopure water. DNA sequencing was performed by Genewiz (South Plainfield, New Jersey). Sequence assembly and analysis of chromatograms were performed with Geneious prime version 2023.2.1. Forward and reverse sequences were aligned using MAFFT tool (Katoh and Standley, 2013) and low-quality read-ends were trimmed. All sequence data were deposited in GenBank.

### Phylogenetic analysis

Using the amplified 28S and ITS2 sequences, we performed maximum likelihood (ML) and Bayesian inference (BI) analysis. The ITS2 inferred phylogenetic tree did not show enough resolution, perhaps because of low taxon sampling, and therefore is not shown herein. The ingroup taxa of the 28S analysis comprised our newly generated sequences and those ascribed to Diplodiscidae, Cladorchiidae Fischoeder, 1901, and Zonocotylidae Yamaguti, 1963, following Pantoja et al. (2019), Queiroz et al. (2021), Bedin et al. (2024), and Uribe et al. (2025), but excluding sequences shorter than 1000 base pairs (bp). The outgroup taxa include four sequences ascribed to Zygocotylidae Ward, 1917, Gastrodiscidae Monticelli, 1892, and Paramphistomidae Fischoeder, 1901, following tree topologies of Paramphistomoidea Fischoeder, 1901 recovered by Alves et al. (2020) and Bedin et al. (2024). Sequences were aligned using MAFFT tool (Katoh and Standley, 2013), and the alignment was trimmed to the shortest sequence (1072 bp). ML phylogeny was inferred using IQTREE v.1.16.12 (Nguyen et al., 2015). Substitution model testing was done with ModelFinder (Kalyaanamoorthy et al., 2017) as implemented in IQTREE. After model testing, tree inference was done using best-fitting substitution models (Chernomor et al., 2016). Default tree search parameters were used, except perturbation strength was set to 0.2 and 500 iterations had to be unsuccessful to stop the tree search. Tree inference was done 20 times with only the tree with the best log-likelihood score reported. Support for relationships was measured with 1000 ultra-fast bootstrap replicates (UFBoot) (Hoang et al., 2018). To perform BI analysis, the aligned sequences were reformatted (from .fasta to .nexus) using the web application ALTER (Glez-Peña et al. 2010). BI was run using MrBayes version 3.2.7a (Ronquist and Huelsenbeck, 2003) using substitution model averaging (nst-mixed) and a gamma distribution to model rate-heterogeneity. Defaults were used in all other parameters. Three independent runs with four Metropolis-coupled chains were run for 5000000 generations, sampling the posterior distribution every 1000 generations. Convergence was checked using Tracer v1.6.1 and the ‘sump’ command in MrBayes: all runs appeared to reach convergence after discarding the first 25% of generations as burn-in. A majority-rule consensus tree of the post-burn posterior distribution was generated with the ‘sumt’ command in MrBayes. The inferred phylogenetic trees were visualized using FigTree v1.4.4 (Rambaut et al., 2018) and further edited for visualization purposes with Adobe Illustrator (Adobe Systems).

## Results

### Taxonomic summary

Paramphistomoidea Fischoeder, 1901

Diplodiscidae Cohn, 1904

*Catadiscus* Cohn, 1904

**Fig. 1:**
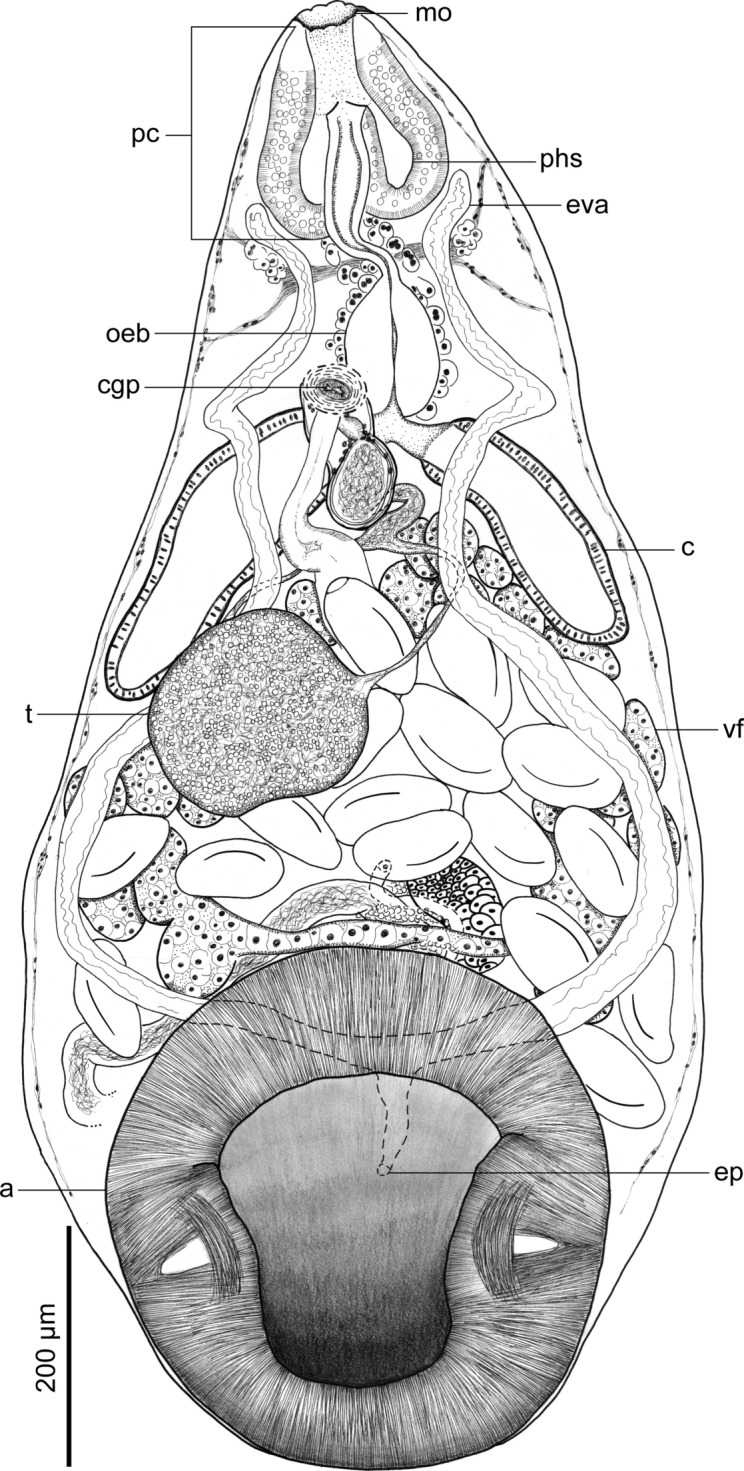
*Catadiscus marielaosornae* Cajiao-Mora and Bullard **n. sp.** (Digenea: Diplodiscidae) (holotype USNM 1694133) infecting the intestine of the Peters’ thin-toed frog, *Leptodactylus petersii* (Steindachner) (Anura: Leptodactylidae) from the Yahuarcaca Lake System (Leticia, Amazonas, Colombia). Ventral view. Scale view beside bar. Abbreviations: acetabulum (a); caecum (c); common genital pore (cgp); excretory pore (ep); excretory vesicle arm (eva); mouth (mo); oesophageal bulb (oeb); pharyngeal complex (pc); pharyngeal sac (phs); testis (t); vitelline follicle (vf)

**Fig. 2:**
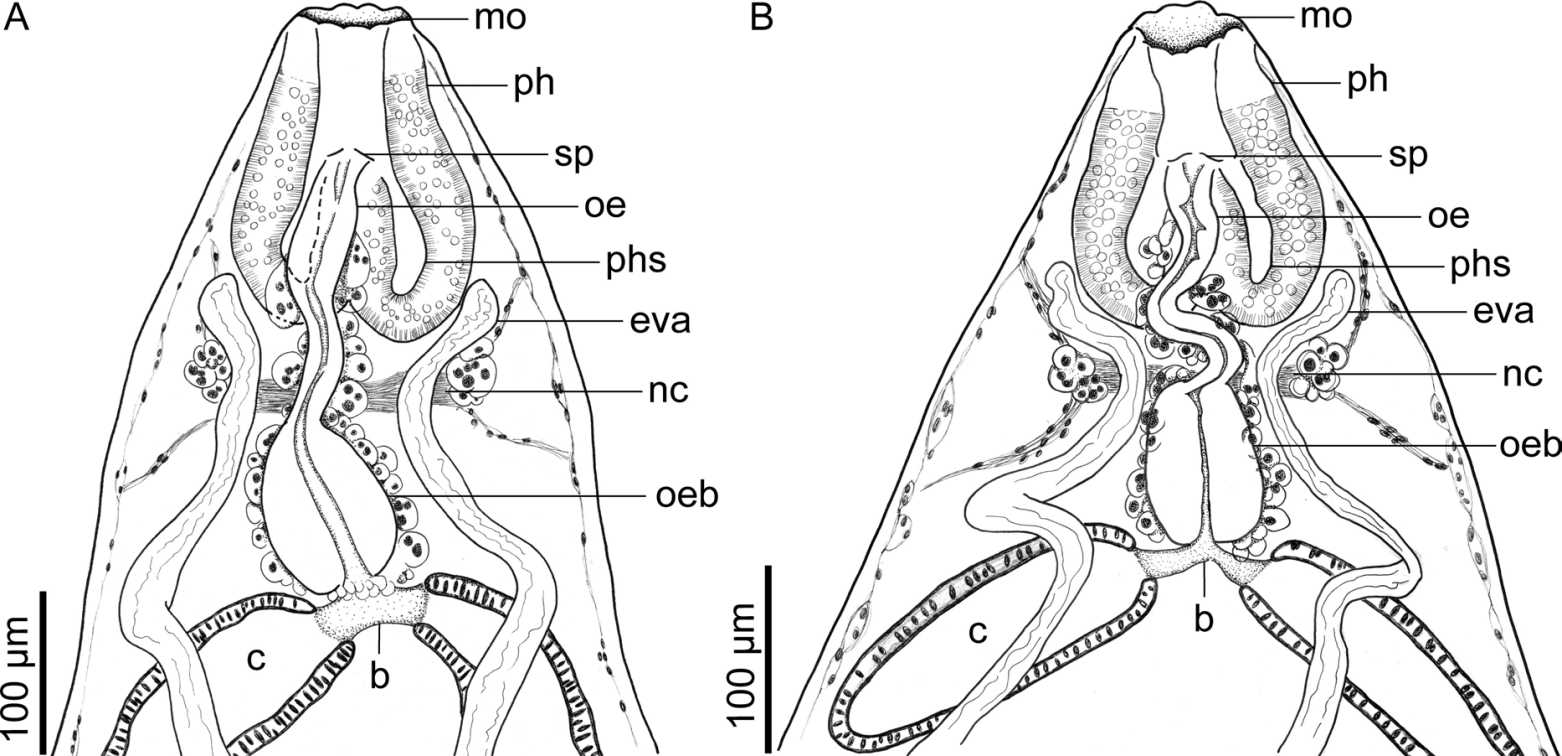
*Catadiscus marielaosornae* Cajiao-Mora and Bullard **n. sp.** (Digenea: Diplodiscidae) infecting the intestine of the Peters’ thin-toed frog, *Leptodactylus petersii* (Steindachner) (Anura: Leptodactylidae) from the Yahuarcaca Lake System (Leticia, Amazonas, Colombia) showing intra-specific variation. Scale view beside bars. **A** Anterior end of paratype USNM 1694134, ventral view. Terminal genitalia and common genital pore are not shown. **B** Anterior end of paratype 349-PA-FCA-UdeA, ventral view. Terminal genitalia and common genital pore are not shown. Abbreviations: caecum (c); excretory vesicle arm (eva); mouth (mo); nerve commissure (nc); oesophageal bifurcation (b); oesophageal bulb (oeb); oesophagus (oe); pharyngeal sacs (phs); pharynx (ph); upper oesophageal sphincter (sp)

**Fig. 3:**
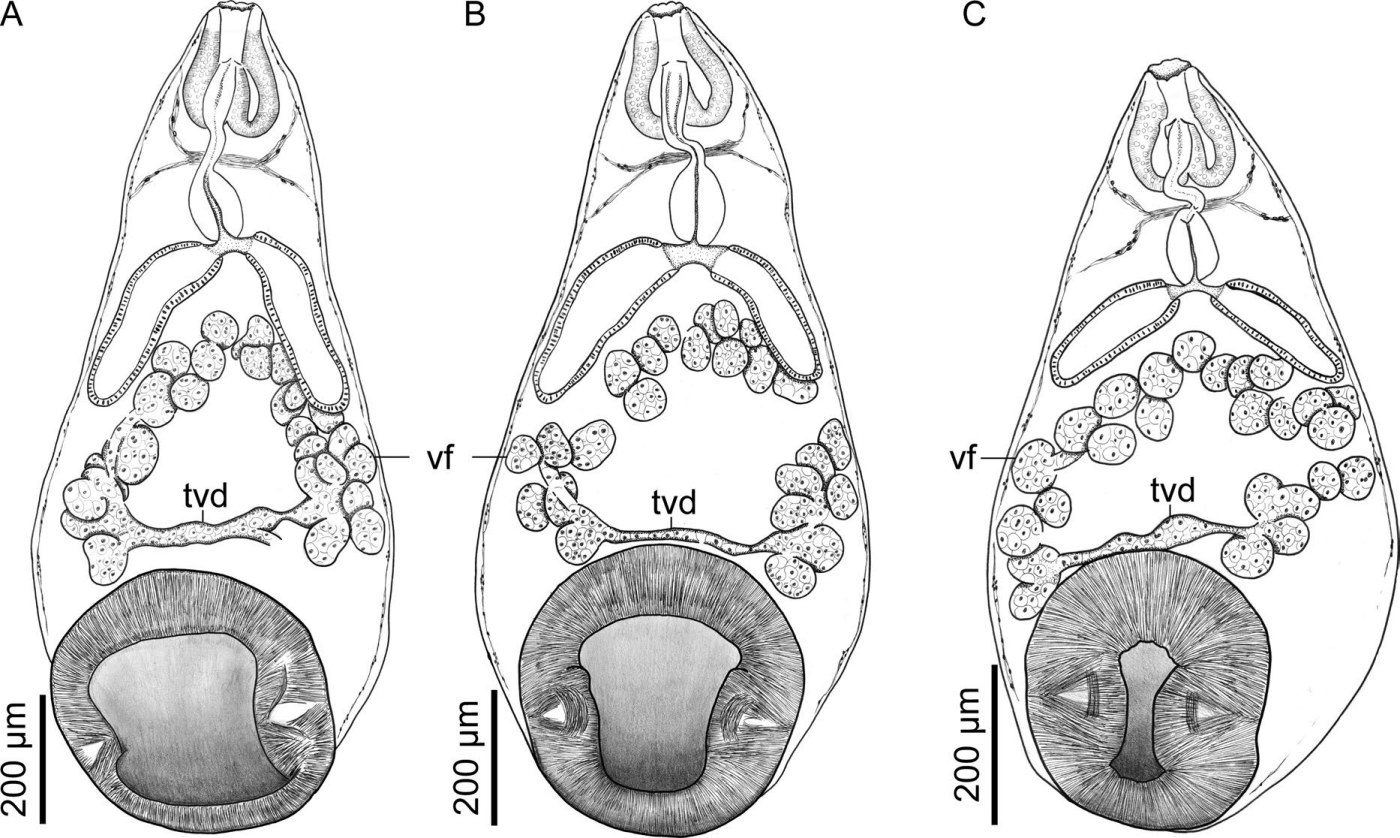
*Catadiscus marielaosornae* Cajiao-Mora and Bullard **n. sp.** (Digenea: Diplodiscidae) infecting the intestine of the Peters’ thin-toed frog, *Leptodactylus petersii* (Steindachner) (Anura: Leptodactylidae) from the Yahuarcaca Lake System (Leticia, Amazonas, Colombia) showing intra-specific variation. Scale view beside bars. **A** Paratype USNM 16941134, ventral view. Male and female genitalia are not shown. **B** Holotype USNM 1694133, ventral view. Male and female genitalia are not shown. **C** Paratype 349-PA-FCA-UdeA, ventral view. Male and female genitalia are not shown. Abbreviations: transverse vitelline duct (tvd); vitelline follicle (vf)

**Fig. 4:**
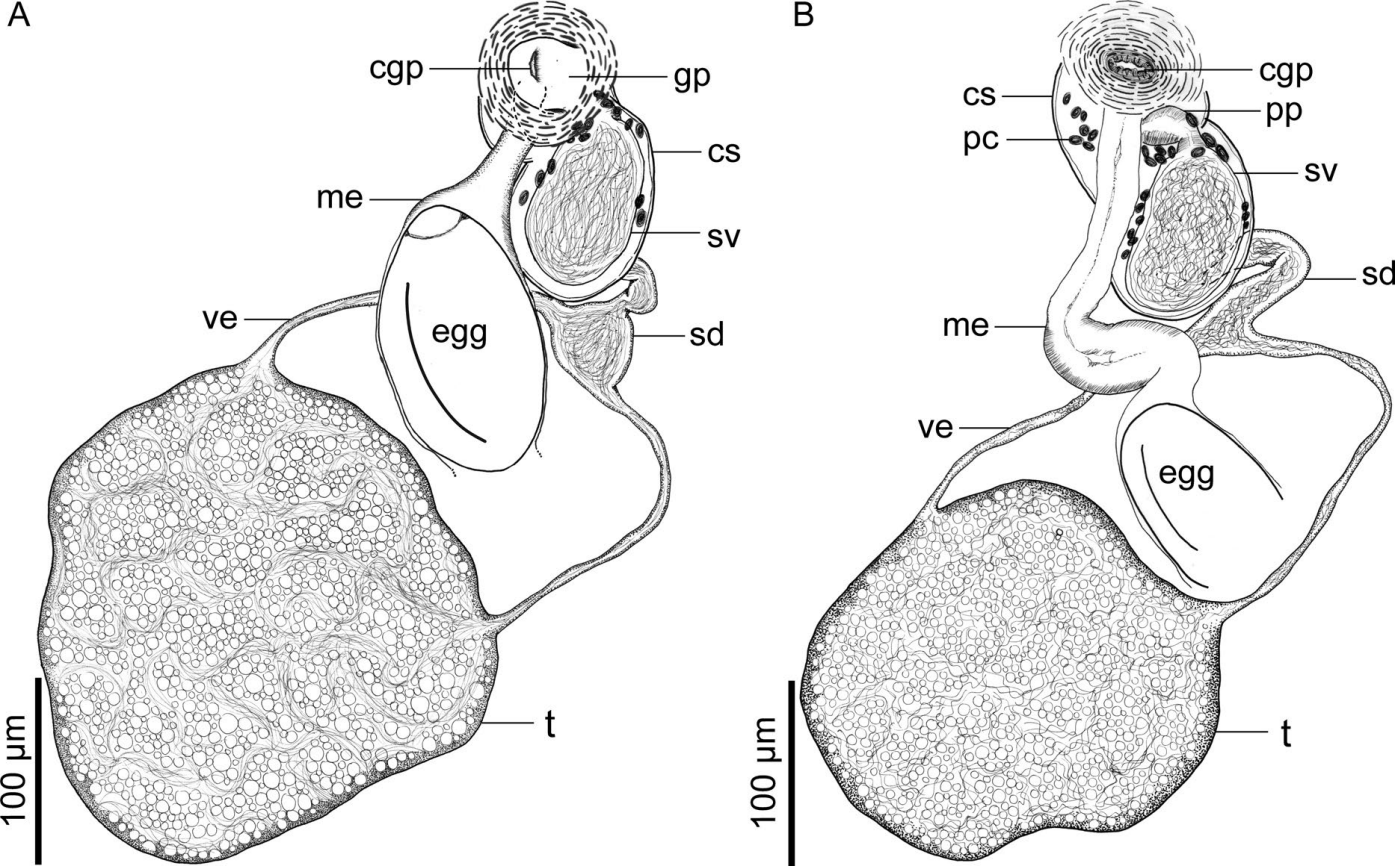
*Catadiscus marielaosornae* Cajiao-Mora and Bullard **n. sp.** (Digenea: Diplodiscidae) infecting the intestine of the Peters’ thin-toed frog, *Leptodactylus petersii* (Steindachner) (Anura: Leptodactylidae) from the Yahuarcaca Lake System (Leticia, Amazonas, Colombia) showing intra-specific variation. Scale view beside bars. **A** Paratype USNM 1694134, ventral view. Male genitalia and female terminal genitalia. **B** Holotype USNM 1694133, ventral view. Male genitalia and female terminal genitalia. Abbreviations: cirrus sac (cs); common genital pore (cgp); genital papillae (gp); metraterm (me); prostatic cells (pc); pars prostatica (pp); seminal duct (sd); seminal vesicle (sv); testis (t); vas efferent (ve)

**Fig. 5:**
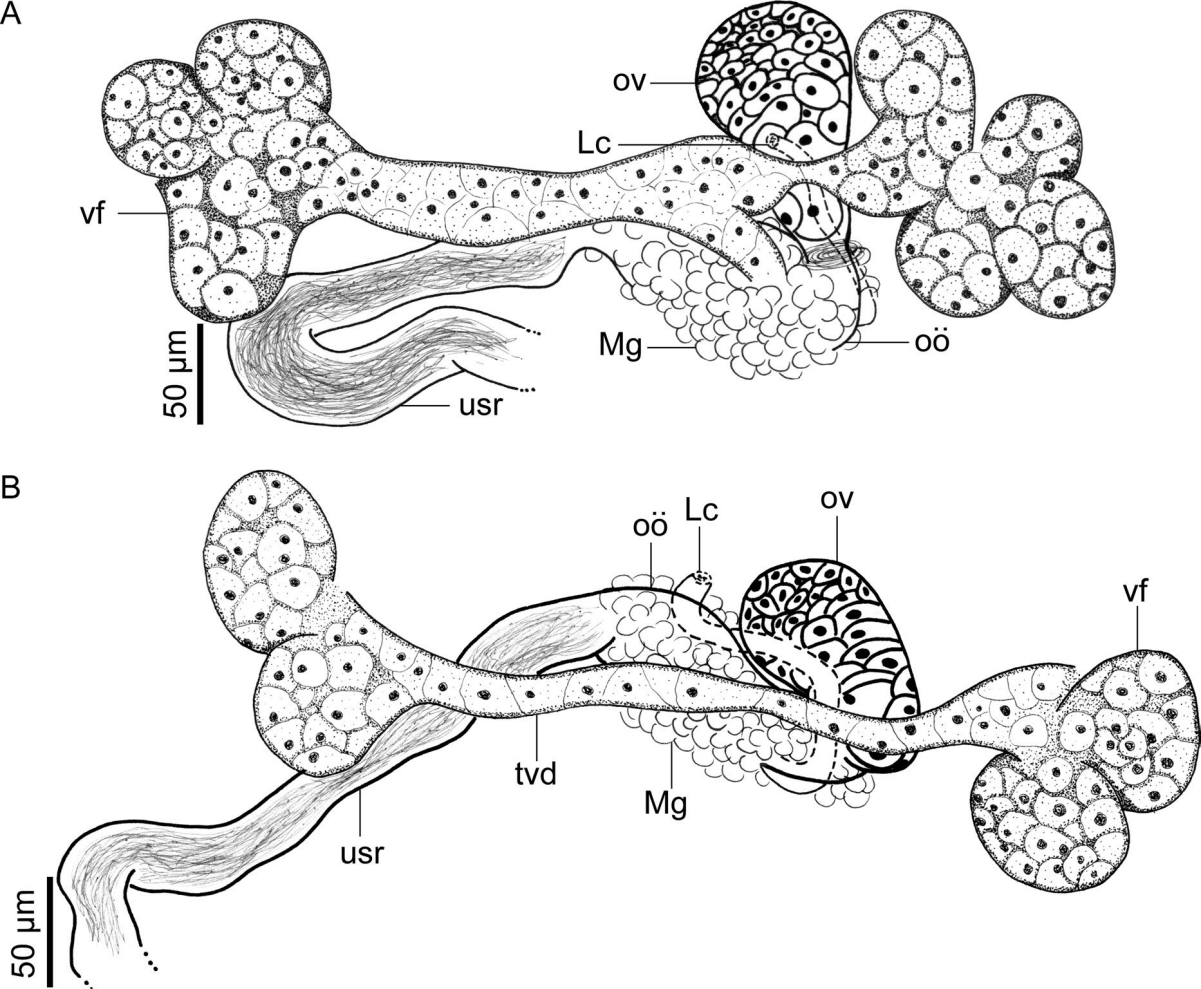
*Catadiscus marielaosornae* Cajiao-Mora and Bullard **n. sp.** (Digenea: Diplodiscidae) infecting the intestine of the Peters’ thin-toed frog, *Leptodactylus petersii* (Steindachner) (Anura: Leptodactylidae) from the Yahuarcaca Lake System (Leticia, Amazonas, Colombia) showing intra-specific variation. Scale view beside bars. **A** Paratype USNM 1694134, ventral view. Female genitalia. **B** Holotype USNM 1694133, ventral view. Female genitalia. Abbreviations: Laurer’s canal (Lc); Mehlis’ gland (Mg); oötype (oö); ovary (ov); transverse vitelline duct (tvd); uterine seminal receptacle (usr); vitelline follicle (vf)

**Fig. 6:**
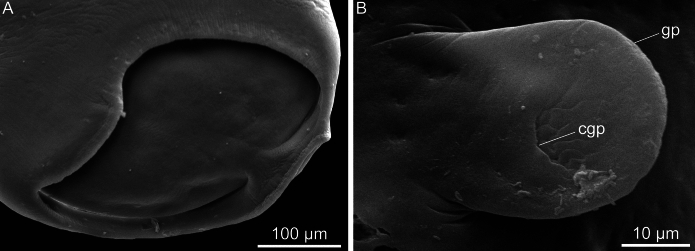
Scanning electron microcopy of *Catadiscus marielaosornae* Cajiao-Mora and Bullard **n. sp.** (Digenea: Diplodiscidae) infecting the intestine of the Peters’ thin-toed frog, *Leptodactylus petersii* (Steindachner) (Anura: Leptodactylidae) from the Yahuarcaca Lake System (Leticia, Amazonas, Colombia). Scale view beside bars. **A** Acetabulum. **B** Genital papilla. Abbreviations: common genital pore (cgp); genital papilla (gp)

*Catadiscus marielaosornae* **Cajiao-Mora and Bullard** **n. sp.** (Figs. 1, 2, 3, 4, 5 and 6) (Table 3).

**Table 3:** Measurements from the type descriptions of *Catadiscus* Cohn, 1904 (Digenea: Diplodiscidae) species infecting ditch frogs (*Leptodactylus* Fitzinger; Anura: Leptodactylidae)

Species	*Catadiscus marielaosornae* Cajiao-Mora and Bullard n. sp.	*Catadiscus marinholutzi* Freitas and Lent, 1939	*Catadiscus eldoradiensis* Artigas and Pérez, 1964	*Catadiscus uruguayensis* Freitas and Lent, 1939	*Catadiscus inopinatus* Freitas, 1941	*Catadiscus rodriguezi* Caballero, 1955
Host	Peters’ thin-toed frog, *Leptodactylus petersii* Steindachner (Anura: Leptodactylidae)	criolla frog, *Leptodactylus latrans* (Steffen) (Anura: Leptodactylidae)	criolla frog	criolla frog	criolla frog	South American bullfrog, *Leptodactylus pentadactylus* (Laurenti) (Anura: Leptodactylidae)
Locality	Yahuarcaca Lake System, Amazonas, Colombia	Mato Grosso, Brazil	Eldorado, São Paulo, Brazil	Montevideo, Uruguay	Brazil; Paraguay	El Valle de Antón, Panamá
Source	Present study	Freitas and Lent (1939)	Artigas and Perez (1964)	Freitas and Lent (1939)	Freitas (1941)	Caballero (1955)
N	3	8	4	22	5	20
Total body length	965–1241 (1141; 3)	1610–2490	2160–3000	960–2160	2510–3950	1245–1311
Forebody length	623–845 (750; 3)	–	–	–	–	–
Body maximum width	517–587 (553; 3)	1050–1290	800–1040	450–710	1210–1710	365–548
Width at oesophageal bifurcation	318–353 (332; 3)	180–220	–	–	–	–
Width at pharyngeal sacs	186–228 (209; 3)	–	–	–	–	–
Width at testis	324–501 (433; 3)	–	–	–	–	–
Acetabulum length	345–422 (393; 3)	700–900	600–750	400–610	650–880	365–465
Acetabulum width	307–424 (349; 3)	530–980	560–870	310–560	530–780	382–418
Pharyngeal complex total length	166–179 (172; 3)	200–220*	200–310*	180–230*	261–278*	–
Anterior pharyngeal complex width	91–106 (100; 3)	–	–	–	–	–
Posterior pharyngeal complex width	135–156 (146; 3)	–	–	130–220*	240–320*	–
Pharyngeal wall thickness	27–36 (32; 3)	–	–	–	–	–
Anterior pharyngeal lumen length	70–81 (77; 3)	–	100–180*	–	96–113*	–
Anterior pharyngeal lumen width	29–37 (34; 3)	–	–	–	–	–
Pharyngeal sacs length	92–116 (104; 3)	–	100–130*	–	–	186–190*
Pharyngeal sacs width	62–80 (74; 3)	–	–	–	–	65–76*
Pharyngeal sacs lumen length	65–90 (77; 3)	–	–	–	–	–
Pharyngeal sacs lumen width	19–30 (23; 3)	–	–	–	–	–
Oesophagus length (sphincter to oesophageal bifurcation)	215–288 (263; 3)	430–510*	380–540*	290–350*	520–600*	258–327*
Anterior oesophagus width	24–35 (31; 3)	–	–	–	–	8
Middle oesophagus width	17–21 (18; 3)	–	–	–	–	–
Posterior oesophagus (bulb) length	90–105 (99; 3)	150–180*	130–200*	70–120*	130–170*	106–152*
Posterior oesophagus (bulb) width	64–77 (71; 3)	60–100*	100–180*	50–100*	90–110*	61–118*
Oesophagus bifurcation to anterior body end length	288–371 (338; 3)	–	–	–	–	–
Dextral caecum length	199–311 (268; 3)	–	510–800	–	–	243–312 (caeca)
Dextral caecum width	67–96 (81; 3)	–	100–180	–	–	76–91 (caeca)
Dextral caecum wall thickness	12–13 (13; 3)	–	–	–	–	–
Sinistral caecum length	186–308 (247; 3)	–	500–670	–	–	–
Sinistral caecum width	56–80 (69; 3)	–	120–160	–	–	–
Sinistral caecum wall thickness	9–13 (11; 3)	–	–	–	–	–
Common genital pore to anterior body end length	262–371 (314; 3)	–	–	–	–	415–448
Cirrus sac length	125–138 (131; 3)	150–180	160–420	100–150	140–190	99–106
Cirrus sac width	52–68 (60; 3)	170–250	110–120	30–70	140–210	49–65
Cirrus sac wall thickness	4–6 (5; 3)	–	–	–	–	–
Seminal vesicle length	77–92 (83; 3)	–	–	–	–	–
Seminal vesicle width	40–46 (42; 3)	–	–	–	–	–
Seminal duct length	98 (1)	–	–	–	–	–
Seminal duct width	18–31 (23; 3)	–	–	–	–	–
Vasa efferentia max width	6–7 (6; 3)	–	–	–	–	–
Testis length	174–235 (201; 3)	280–530	350–600	100–250	430–520	133–160
Testis width	162–225 (201; 3)	330–550	220–410	150–280	430–480	167–198
Ovary length	104–128 (116; 2)	100–170	100–200	80–170	170–230	38–53
Ovary width	59–74 (67; 2)	120–150	80–140	70–80	160–170	114–122
Ovary to anterior body end length	558–720 (657; 3)	–	–	–	–	–
Transverse vitelline duct length	298–304 (271; 3)	–	–	–	–	–
Uterus length (length of body occupied by uterus)	487–594 (543; 3)	–	–	–	–	–
Uterus width (width of body occupied by uterus)	470–532 (506; 3)	–	–	–	–	–
Eggs length	109–127 (118; 5)	113–126	60–82	105–113	84–101	106–118
Eggs width	55–72 (64; 5)	59–71	30–32	53–55	50–55	68–72
Excretory pore to anterior body end length	806–991 (919; 3)	–	–	–	–	–
Excretory vesicle arm to anterior body end length	145–167 (157; 3)	–	–	–	–	–
Excretory vesicle arms max width	27–32 (29; 3)	–	–	–	–	–

*Estimated from published drawings and measurements

*Type-host:* Peters’ thin-toed frog, *Leptodactylus petersii* (Steindachner) (Anura: Leptodactylidae) (vouchers SINCHI-A-10853–10856, and SINCHI-A-10884)

*Type-locality:* Yahuarcaca Lake System, 4°11′36.7″S, 69°57′17″W (Leticia, Amazonas, Colombia).

*Site of infection:* Intestine.

*Prevalence and intensity:* four of five adult *L. petersii* were infected with 19 adult specimens of *C. marielaosornae*. The heaviest infection was one frog infected with 10 adult digeneans while the lowest one were two frogs infected with one adult digenean each.

*Type-specimens:* holotype USNM 1694133; paratypes USNM 1694134, 1694135, 1694136; 348, 349, 350, 351-PA-FCA-UdeA

*ZooBank registration:* urn:lsid:zoobank.org:act:DE93EA6A-81A0-4E69-802C-9A89E3F09A7B

*Representative DNA sequences:* GenBank PX049165, PX049166 (28S); PX049167, PX049168 (ITS2)

*Etymology:* the specific name “*marielaosornae*” honors Mariela Osorno-Muñoz, scientist and curator of the amphibian collection of the SINCHI Institute, for her contributions to the knowledge and conservation of Colombia’s amphibians and reptiles, especially from the Colombian Amazon.

### Description

*Based on five heat-killed, stained, and permanently whole-mounted adults* (*measurements of three specimens in* Table 3):

Body pyriform, 2× longer than wide, widest at anterior margin of acetabulum (Fig. 1). Forebody length 64–68% (66%; 3) of total body length. Tegument smooth, having a papilliform projection in anterior third of body; papilliform projection containing common genital pore (Figs. 1, 4, 6B). Acetabulum rounded in outline (Figs. 1, 3), representing 33–36% (35%; 3) of total body length, sub-terminal to terminal (Fig. 1, 3, 6A), at posterior body end, having a muscular horizontal constriction at midpoint of the vertical axis with reemergent post-constriction margins (Figs. 1, 3, 6A), lacking an accessory sucker. Mouth terminal, at anterior body end, having undulated edges (Figs. 1, 2, 3). Nerve commissure at middle portion of oesophagus, dorsal to oesophagus and excretory vesicle arms (Figs. 1, 2); nerve chords extending from nerve commissure anteriad and posteriad at sides of body (Figs. 1, 2). Pharyngeal complex muscular, at anterior body end, comprising anterior pharynx, upper oesophageal sphincter, and pharyngeal sacs (Figs. 1, 2, 3); anterior pharynx having a wide lumen and muscular walls, continued by oesophagus at midline and pharyngeal sacs laterally (Figs. 1, 2, 3); upper oesophageal sphincter central in pharyngeal complex (Fig. 2); pharyngeal sacs lateral to oesophagus, having muscular wall; wall confluent in midline (Fig. 2). Oesophagus ventral to pharyngeal sacs, medial, slightly sinuous, representing 22–23% (23%; 3) of total body length, having a thick wall, having a wider anterior portion, a thinner middle portion, and a bulb-like posterior portion, bifurcating posterior to the bulb, having associated glandular-like cells in its extension (Figs. 1, 2, 3); oesophageal bulb 2–3× wider than anterior oesophageal portion, 3–5× wider than middle oesophageal portion. Intestinal caeca short, asymmetrical in length (Figs. 1, 3B), extending to equatorial level of body, having an epithelial-like wall (Figs. 1, 2, 3); dextral caecum representing 32–37% (35%; 3) of total body length; sinistral cecum representing 19–25% (21%; 3) of total body length.

Testis single, rounded to irregular in outline (Figs. 1, 4), equatorial, in dextral side of body (Fig. 1), representing 14–19% (20%; 3) of total body length, acetabulum: testis width ratio 1: 2–3. Vasa efferentia emerging from testis at two separated points (Fig. 4), extending anteriad, connected to seminal duct (Fig. 4). Seminal duct enlarged with sperm, slightly convoluted, extending anteriad, connected to seminal vesicle. Cirrus sac dextral to oesophageal bifurcation, ventral to dextral caecum (Fig. 1), having a thin wall (Fig. 4), containing seminal vesicle, pars prostatica, few prostatic cells, and ejaculatory duct (Figs. 1, 4). Seminal vesicle oval (Fig. 4). Pars prostatica tub-like, muscular. Ejaculatory duct extending ventrally into genital papillae, opening into common genital pore. Common genital pore dextral to oesophageal bulb, anterior to oesophageal bifurcation (Fig. 1), located in genital papilla (Fig. 6B); genital papilla protruding from ventral surface of body (Figs. 4, 6B).

Ovary elongated, balloon-like in outline (Fig. 5), post-testicular (Fig. 1), sinistral, dorsal to transverse vitelline duct and uterus (Figs. 1, 5), immediately anterior to acetabulum (Fig. 1). Oviduct emerging from posterior margin of ovary (Fig. 5), short. Laurer’s canal elongate, emerging from oviduct, extending anteriad, sinuous, opening on dorsal body surface (Figs. 1, 5). Oötype surrounded by dense Mehlis’ gland (Fig. 5), continued by uterus seminal receptacle. Uterus convoluted (Fig. 1), extending posteriad to ovary and then anteriad to it, occupying 40–57% (48%; 3) of total body length, having numerous eggs, containing sperm in its proximal portion (Figs. 1, 5), distal portion having a metraterm (Figs. 1, 4); uterus proximal portion serving as a seminal receptacle (Fig. 5). Uterus seminal receptacle elongated, tub-like, extending dextrally and slightly posteriad to vitellarium (Figs. 1, 5). Eggs ovoid, operculate (Fig. 4A). Vitellarium having numerous compact follicles (Fig. 3), confluent anteriad (Fig. 3), connected posteriad by transverse vitelline duct (Figs. 3, 5), dorsal to uterus, testis, and excretory vesicle arms (Fig. 1); vitellarium follicles rounded in outline (Fig. 3), generally same size as ovary; transverse vitelline duct elongated, anteriad to acetabulum (Fig. 3), ventral to uterus seminal receptacle, Mehlis’ gland, and ovary (Fig. 5), connecting with oviduct at an indistinguishable point.

Excretory system tube-like, elongated, having a short stem and long arms (Fig. 1); stem being dorsal to acetabulum, having a dorsal excretory pore, in third quarter of body, bifurcating into excretory vesicle arms (Fig. 1); excretory vesicle arms extending anteriad to pharyngeal complex (Fig. 1), sinuous, ventral to uterus, and caeca.

### Taxonomic remarks

We assigned our specimens to *Catadiscus* Cohn, 1904 because they have a smooth-surfaced, pyriform body (Figs. 1, 3), a centrally-constricted ventro-terminal acetabulum that lacks an accessory sucker (Figs. 1, 3, 6), a pharynx with extramural sacs (Figs. 1–3), an oesophageal bulb (Figs. 1–3), and a single testis.

The new species differs from all but *Catadiscus rodriguezi* Caballero, 1955, *Catadiscus marinholutzi* Freitas and Lent, 1939, and *Catadiscus propinquus* Freitas and Dobbin, 1956 by having a vitellarium that is confluent anteriorly (*vs*. distributed in two non-confluent, lateral fields). It differs from the aforenamed three congeners by having i) a ventral common genital pore that opens anterodextral (*vs*. posterior) to the oesophageal bifurcation, ii) round (*vs*. irregular-shaped) vitelline follicles that become confluent anterior to (*vs*. confluent both anteriad and posteriad to) the testis, and iii) a 1: 2–3 (*vs*. 1: 1.3–1.5 [*C. marinholutzi*, *C. propinquus*]) ratio of acetabulum width: testis width. The new species further differs from *C. marinholutzi* by being shorter and thinner (Table 3) as well as having a larger testis (Table 3).

### Phylogenetic results

The two amplified 28S (both 1634 bp) and ITS2 (both 405 bp) sequences of *C. marielaosornae* were respectively identical (GenBank accession Nos. PX049165, PX049166 [28S]; PX049167, PX049168 [ITS2]). The 28S sequences of *C. marielaosornae* were most similar to sequences ascribed to adult *C. marinholutzi* infecting the pointedbelly frog, *Leptodactylus podicipinus* (Cope) (Anura: Leptodactylidae) (GenBank accession No. MW618662) and sequences ascribed to cercariae of *C. marinholutzi* infecting a planorbid snail, *Drepanotrema lucidum* (Pfeiffer) (Gastropoda: Planorbidae) (GenBank accession No. MW618663) from Mato Grosso do Sul, Brazil (Queiroz et al., 2021). The 28S sequences of *C. marinholutzi* were 1219 bp long and exhibit 99.7% pairwise identity (5–6 bp different; 74% sequence coverage) to our aligned sequences of the new species.

**Fig. 7:**
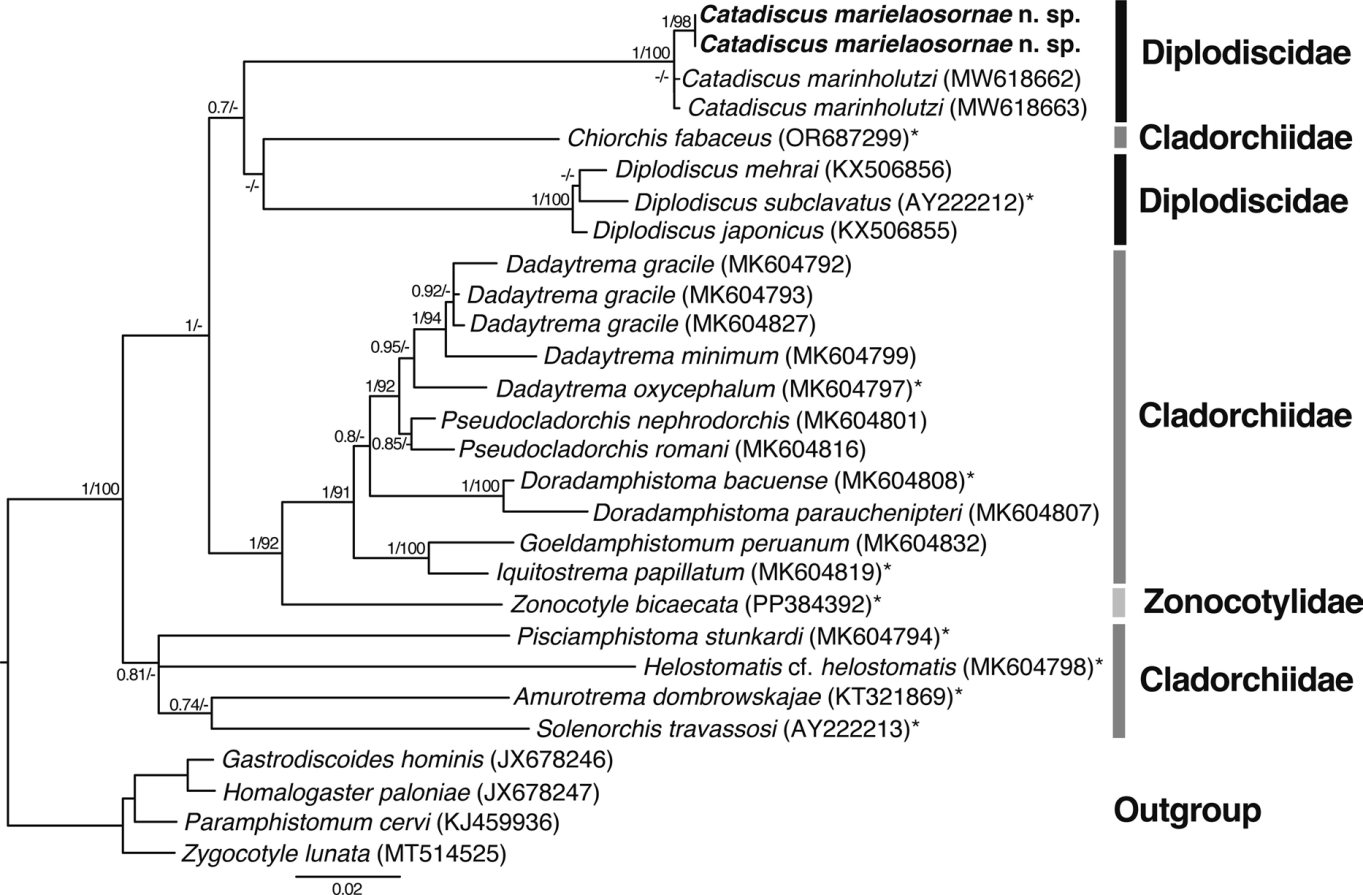
28S maximum likelihood phylogenetic analysis inferred tree. Values aside nodes are posterior probability obtained with BI and bootstrap percentage obtained with ML, respectively. Only those values >0.7 (BI) and >90 (ML) are shown. Scalebar is in substitutions per site. GenBank accession numbers are in parenthesis following each taxon. Type species are indicated by asterisk (*). The newly generated sequences of *Catadiscus marielaosornae* Cajiao-Mora and Bullard **n. sp.** (Digenea: Diplodiscidae) are in bold (GenBank accession Nos. PX049165, PX049166 [28S])

The 28S alignment for the phylogenetic analyses included 28 sequences. After trimming, the alignment was 1072 bp long. The ML and BI inferred trees recovered both Diplodiscidiae and Cladorchiidae as paraphyletic. We recovered our *C. marielaosornae* sequences sister to those of *C. marinholutzi*. Those sequences represent the only sequences of species of *Catadiscus* available to date. *Catadiscus* clade was recovered sister to a weakly supported clade (0.7 posterior probability [BI]; 53 bootstrap [ML]) containing sequences representing species of Cladorchiidae and Diplodiscidae (Fig. 7): *Chiorchis fabaceus* (Diesing, 1838) Fischoeder, 1901 (Cladorchiidae) (GenBank accession No. OR687299) collected from an Antillean manatee, *Trichechus manatus manatus* (Linnaeus) (Sirenia: Trichechidae), from Colombia (Uribe et al., 2025); *Diplodiscus japonicus* Yamaguti, 1936 and *Diplodiscus mehrai* Pande, 1937 (Diplodiscidae) (GenBank accession Nos. KX506855, KX506856, respectively) collected from the Dybowski’s frog, *Rana dybowskii* Günther (Anura: Ranidae), from Russia (Besprozvannykh et al., 2018); and *Diplodiscus subclavatus* (Goeze, 1782) Diesing, 1836 (type-species) (GenBank accession No. AY222212), collected from the marsh frog, *Pelophylax ridibundus* (Pallas) (Anura: Ranidae) from Bulgaria (Olson et al., 2003).

None of those sequences have a voucher specimen but brief descriptions were provided for *C. fabaceus*, *D. japonicus*, and *D. mehrai*. No such description or voucher exist for the sequence ascribed to *D. subclavatus*.

## Discussion

The pharyngeal complex, oesophagus, and oesophageal bulb have been misinterpreted in previous descriptions of species of *Catadiscus*. Jones (2005b) suggested that this is because early workers relied on the traditional morphological characters applied throughout the Digenea.

In specific, the pharyngeal complex was misinterpreted as being an oral sucker with two diverticula; the oesophageal bulb was misinterpreted as the pharynx; the oesophagus was misinterpreted as the pre-pharynx; and usually the oesophagus was described as absent (Cohn, 1903; Travassos, 1926; Lutz, 1928; Freitas and Lent, 1939; Freitas, 1941, 1943; Ruiz, 1943; Mañé-Garzón, 1958; Artigas and Pérez, 1964; Poumarau, 1965; Correa and Artigas, 1978; Incorvaia, 1983; Hamann, 1992). Näsmark (1937), using histological sections of paramphistomes, concluded that the so-called ‘oral sucker’ is a muscular pharynx with two pharyngeal sacs and that the oesophagus had a distal “thickness” or “bulb” (Näsmark, 1937). However, his work was done with mammalian-infecting paramphistomes. Perhaps because of this, or perhaps because they were not aware of the published work of Näsmark (1937), parasitologists working with amphibian+reptile-infecting species of *Catadiscus* did not follow his interpretations (Freitas and Lent, 1939; Freitas, 1941, 1943; Ruiz, 1943; Mañé-Garzón, 1958; Artigas and Pérez, 1964; Poumarau, 1965; Correa and Artigas, 1978; Incorvaia, 1983; Hamann, 1992). But Näsmark’s (1937) approach nevertheless is now applied to those paramphistomes infecting lower vertebrates (Jones, 1991; Jones and Khalil, 1990; Sey, 1991; Jones, 2005a; Queiroz et al., 2021) and we follow it herein. Because of those morphological misinterpretations, previous descriptions of *Catadiscus* spp. include measurements that should be interpreted in their context and carefully.

Freitas and Dobbin (1956; page 440) described the testis of *C. propinquus* as “*deslocado para o lado*” (“*moved to a side*”) but did not specify its dextral/sinistral position nor did they state the dorsal/ventral orientation of the illustrated specimen. Hamann (2004) studied paramphistomes that she identified as *C. propinquus* infecting the Uruguay harlequin frog, *Pseudis limellus* (Cope) (Hylidae) from Argentina. She drew the testis (typically) as positioned along the midline and (less common) slightly dextral (Hamann, 2004; Figure 1; page 31). A critical study and redescription of type-specimens in the Coleção Helmintológica do Instituto Oswaldo Cruz (CHIOC; Rio de Janeiro, Brazil) is needed to confirm the position of the testis in *C. propinquus*.

Details of the female genitalia, genital papilla, and excretory pore were incomplete or lacking entirely for all previous species descriptions for species of *Catadiscus*. Numerous authors generally detail the shape and position of the ovary (Cohn, 1903; Travassos, 1926; Lutz, 1928; Freitas and Lent, 1939; Freitas, 1941, 1943; Ruiz, 1943; Mañé-Garzón, 1958; Artigas and Pérez, 1964; Poumarau, 1965; Correa and Artigas, 1978; Incorvaia, 1983; Hamann, 1992). The description herein comprises the first to detail the oviduct, Laurer’s canal, oötype, uterine seminal receptacle, metraterm, and position of the excretory pore. Also noteworthy is that we confirm the presence of a genital papilla, like in *C. marinholutzi* (see Queiroz et al., 2021), using SEM as well as in the whole mounted specimens. We suspect that a genital papilla is present in several other species of *Catadiscus* but so far has been overlooked. Our SEM images also reveal the musculature in the acetabulum that causes the middle constriction (Fig. 6), which is a diagnostic character for *Catadiscus*.

Diplodiscidae *sensu* Jones (2005a) includes 6 genera (all having a single testis): *Diplodiscus* (type-genus), *Progonimodiscus* Vercammen-Grandjean, 1960; monotypic *Dermatemytrema* Price, 1937; monotypic *Pseudodiplodiscus* Manter, 1962; monotypic *Australodiscus* Sey, 1983; and *Catadiscus* (Jones, 2005a for taxonomic history). Queiroz et al. (2021) asserted that Diplodiscidae was paraphyletic because of the low support for the clade containing *Diplodiscus* and *Catadiscus*, plus a high intergeneric divergence between their 28S sequences of *C. marinholutzi* and those ascribed to *Diplodiscus*. We conclude the same based on the new sequences herein (Fig. 7). Queiroz et al. (2021) suggested that Catadiscinae *sensu* Yamaguti (1971; p 393) (differentiated from other diplodiscids by having a middle acetabular constriction; not accepted in Jones, 2005a, 2005b), should be elevated to family and that it should accommodate *Catadiscus*. Based on the current evidence, we refrain from making taxonomic changes, as we think that additional nucleotide sequences linked to morphological descriptions and/or museum curated vouchers are necessary to advance our understanding of the evolution of Diplodiscidae. The group is woefully under sampled regarding nucleotide sequences: only 2 (*Catadiscus* and *Diplodiscus*) of the 6 recognized genera have representative sequences.

The low support values in our phylogenetic analysis probably are probably because of low taxon sampling. Our results also indicate the need for more and better morphological (diagnostic) features that define and relate genera across families of the Paramphistomoidea, as suggested by single-gene phylogenetic analyses (Queiroz et al., 2021; Montes et al., 2023; Bedin et al., 2024; present study).

A strong biogeographic/continental pattern exists among the morphologically diagnosed genera of Diplodiscidae. Two diplodiscid groups can be identified based on morphology and geographic distribution. First are those species lacking a central accessory sucker or peduncle in the acetabulum and that have been described as maturing in aquatic/semi-aquatic vertebrates in the Americas: *Catadiscus* (infecting amphibians and reptiles), *Dermatemytrema* (infecting turtles), and *Pseudodiplodiscus* (infecting fish) (Jones, 2005a). Second are those species having a central accessory sucker or peduncle in the acetabulum and that have been described as maturing in frogs, amphibians, and reptiles from Africa, Asia, Oceania, or Europe: *Progonimodiscus*, *Australodiscus* (both infecting frogs), and *Diplodiscus* (infecting amphibians and reptiles) (Jones, 2005a). To further evaluate the relevance of the acetabulum morphology to assess systematic classification, nucleotide sequences linked to morphological descriptions and/or museum curated vouchers are indispensable.

## Data Availability

No datasets were generated or analysed during the current study.
